# Financial Burden of Postoperative Adverse Events Following Lobectomy: Cost Analysis From 10 High-Volume Canadian Hospitals

**DOI:** 10.1093/ejcts/ezag008

**Published:** 2026-01-07

**Authors:** Daniel G Jones, Caitlin Anstee, Kazuhiro Yasufuku, Richard Malthaner, Najib Safieddine, Christian Finley, Biniam Kidane, Danny French, Brian Johnston, Lorenzo Ferri, Andrew J E Seely

**Affiliations:** Thoracic Surgery, University of Ottawa, Ottawa, ON, K1H 8L6, Canada; Surgery, Ottawa Health Research Institute, Ottawa, ON, K1H 8L6, Canada; Surgery, Ottawa Health Research Institute, Ottawa, ON, K1H 8L6, Canada; Thoracic Surgery, University of Toronto, Toronto, ON, M5G 2C4, Canada; Thoracic Surgery, London Health Sciences Centre, London, ON, N6A 5A5, Canada; Thoracic Surgery, Michael Garron Hospital, Toronto, ON, M4C 3E7, Canada; Thoracic Surgery, St Joseph’s Healthcare Hamilton, Hamilton, ON, L8N 1Y3, Canada; Thoracic Surgeon, University of Manitoba, Winnipeg, MB, E2L 4L2, Canada; Thoracic Surgeon, Dalhousie University, Halifax, NS, B3H 2Y9, Canada; Thoracic Surgeon, St-John Regional Hospital, Saint John, NB, E2L 4L2, Canada; Thoracic and Upper GI, McGill University, Montreal, QC, H3G 1A4, Canada; Thoracic Surgery, University of Ottawa, Ottawa, ON, K1H 8L6, Canada; Surgery, Ottawa Health Research Institute, Ottawa, ON, K1H 8L6, Canada

**Keywords:** index hospital costs, postoperative adverse events, lung cancer, lobectomy

## Abstract

**Objectives:**

Lobectomy remains a cornerstone of curative intent treatment for lung cancer; however, postoperative adverse events (AEs) remain common, harmful, and costly. To support value-based quality improvement (QI) programmes, we sought to estimate the in-hospital costs of AEs following lobectomy and identify which complications are the primary cost drivers.

**Methods:**

Lobectomy data from 10 Canadian hospitals were included (2017-2022). Annual lobectomy volume, demographics, length of stay (LOS), incidence, and severity of AEs were obtained from a prospectively collected national database. Using literature-derived index hospitalization costs of AEs, supported by Canadian Institution of Health Information database, estimates of annual AE costs were obtained (2025 CDN$).

**Results:**

Mean annual lobectomy volume 1150 (SD = 165): 44% male, aged 67 years (SD = 10.9), median LOS of 4 days (interquartile range [IQR] = 4), with minimally invasive surgery performed in 86%. Prolonged air leak (PAL) contributed 51% of total AEs occurrences, followed by atrial arrhythmia (13%), pneumonia (7.9%), reoperation (5.2%), atelectasis (3.9%), delirium (3.4%), transfusion (2.8%), respiratory failure (2.8%), empyema (2.2%), acute kidney injury (1.7%), and pulmonary embolism (1.2%), adding over $7.31 million (M) to hospital-level costs. PAL, mean annual incidence of 17%, was the strongest driver of costs. Extrapolated nationally, lobectomy-related AEs are estimated to contribute over $48 million in excess annual costs.

**Conclusions:**

Postoperative AEs following lobectomy impose substantial financial burdens, with PAL alone accounting for more than half of total costs. These findings underscore the need for value-based QI initiatives targeting high-impact AEs, requiring coordinated action among surgeons, hospital leadership, and policymakers.

## Graphical abstract

**Figure:**
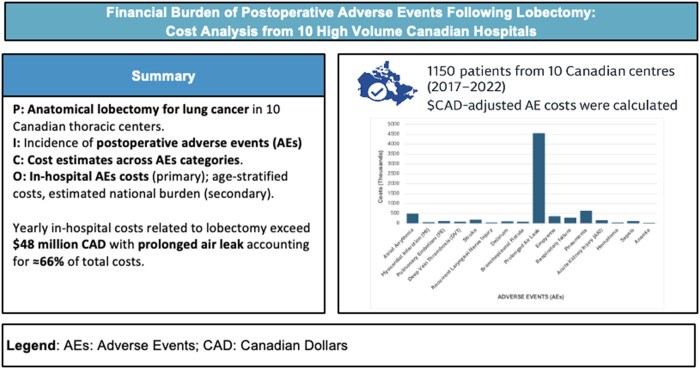

## Introduction

The rising cost of healthcare and the economic burden of adverse events (AEs) following thoracic surgery remains a significant challenge in the delivery of value-based care for patients with lung cancer. Postoperative AEs—defined as deviations from an ideal recovery—are associated with prolonged hospital length of stay (LOS), increased readmissions, higher resource utilization, reduced quality of life, and worsen overall survival.^1–3^ While standardized perioperative protocols and enhanced recovery pathways have been introduced to mitigate these risks, their implementation alone has not eliminated the considerable variability in outcomes or costs across institutions.^4^

In Canada, lung cancer is the leading cause of cancer-related mortality, accounting for approximately 20 700 deaths annually and representing one-quarter of all cancer deaths.^5^ For patients with early-stage disease, anatomical resection via lobectomy remains the standard of care.^6^ Although the clinical risks of lobectomy are well described,^7^^,^^8^ there is a paucity of data on the financial implications of postoperative complications in this setting. Moreover, the absence of standardized cost-reporting frameworks within thoracic surgery has limited our understanding of how specific AEs contribute to in-hospital costs and hindered efforts to benchmark performance and guide quality improvement (QI) initiatives.

A clearer understanding of the cost burden associated with AEs after lobectomy is essential to inform resource allocation, enhance perioperative management, and support the development of cost-effective strategies aimed at reducing complications. The primary objective of this study is to evaluate the direct in-hospital costs associated with AEs following lobectomy. A secondary objective was to assess how the cost burden of AEs varied by age group (<65 vs ≥65 years), given the well-recognized association between advanced age, postoperative morbidity, and healthcare resource utilization.

## Methods

### Data sources

The Canadian Association of Thoracic Surgeons (CATS) database is a national, voluntary, prospectively collected, externally audited database. The CATS database collects patient demographics as well as all postoperative AEs data. From January 2017 to December 2022, data from all patients who had undergone a lobectomy for lung cancer were included, encompassing data from 10 high-volume Canadian lung cancer centres. Other data sources included Canadian Institute of Health Information (CIHI), a nationally governed database describing annual surgical volumes across Canada. The study population included all patients who had undergone a lobectomy for lung cancer between January 2017 and December 2022. Individual hospital lobectomy annual volume, patient demographics (age, % male/female), diagnosis, LOS, surgical procedure (% minimally invasive surgery, MIS), and postoperative AEs were assessed. Prolonged air leak (PAL) was defined as an air leak persisting beyond postoperative day 5, in accordance with the Society of Thoracic Surgeons (STS) and European Society of Thoracic Surgeons (ESTS) definitions. This standardized definition is used across all centres contributing to the CATS database. The unit of analysis in this study was the individual patient. For each centre, the incidence of AEs was calculated as the number of patients experiencing the AE divided by the total number of lobectomy cases performed. National incidence rates were derived from pooled patient-level data across all centres. For inter-institutional comparisons, mean incidence rates, standard deviation (SD), and coefficient of variation (CoV) were calculated and weighted by each centre’s annual lobectomy volume. For centre-level comparisons, data are presented as median and interquartile range (IQR) to reflect the small number of centres (*n* = 10) and potential non-normality of distributions. Cost estimates for specific AEs were obtained from the literature-derived index hospitalization cost of AEs.^9^ All cost estimates are valued in Canadian dollars (CDN) adjusted to the present year’s value (CDN 2025).

### Ethics approval

The risk of privacy breach was mitigated by anonymizing and de-identifying data, and no consent was required. The study was approved by the Ottawa Hospital Research Institute REB [Protocol #: 20220685-01H].

### Statistical analysis

Descriptive statistics were used to describe patient and surgical data. Categorical data were expressed as frequencies and percentages. Continuous variables were assessed for normality using the Shapiro-Wilk test and visual inspection of histograms and Q-Q plots. Normally distributed variables were expressed as mean ± SD and compared using the Student’s t-test. Non-normally distributed variables were reported as median with IQR and compared using the Mann-Whitney *U*-test. Missing data were assessed for all baseline demographic and outcome variables. Variables with missing values <5% were analysed using complete case analysis, with denominators adjusted accordingly. No imputation was performed, as missingness was assumed to be random and minimal across centres. Sensitivity analyses confirmed that the exclusion of cases with missing data did not materially alter results. In addition, a prespecified age-stratified analysis was conducted (<65 vs ≥65 years) to evaluate the impact of age on the cost of in-hospital complications. The 65-year threshold was selected because it is the standard definition of older adults in surgical and health-economic literature. Costs were adjusted for inflation to real dollars using the annual consumer price index for each year (CDN 2025). When necessary, values reported in foreign currencies (USD or EUR) were converted to CDN using the average annual exchange rate for the corresponding year. Cost estimates were calculated for each AE independently, using published literature-derived index hospitalization costs multiplied by the observed incidence of each AE. The CATS dataset does not assign costs to individual patients; this approach assumes additive costs across AE categories. Our cost model assumes independence between complications, meaning that patients experiencing multiple AEs contribute to several AE-specific cost categories. This event-based approach may modestly inflate cumulative cost estimates; however, it enables identification of the highest-cost complications and supports prioritization for QI. Our study design does not incorporate multistate modelling or hierarchical adjustment, and results should be interpreted as complication-level cost estimates, not cumulative per-patient costs. Overall institutional cost savings were calculated using the resulting proportional increase in hospital cost along with the estimated reduction in the complication rate. The CIHI database was assessed in the year 2019.^9^ A value of *P* < .05 was considered significant.

## Results

**Figure 1.**
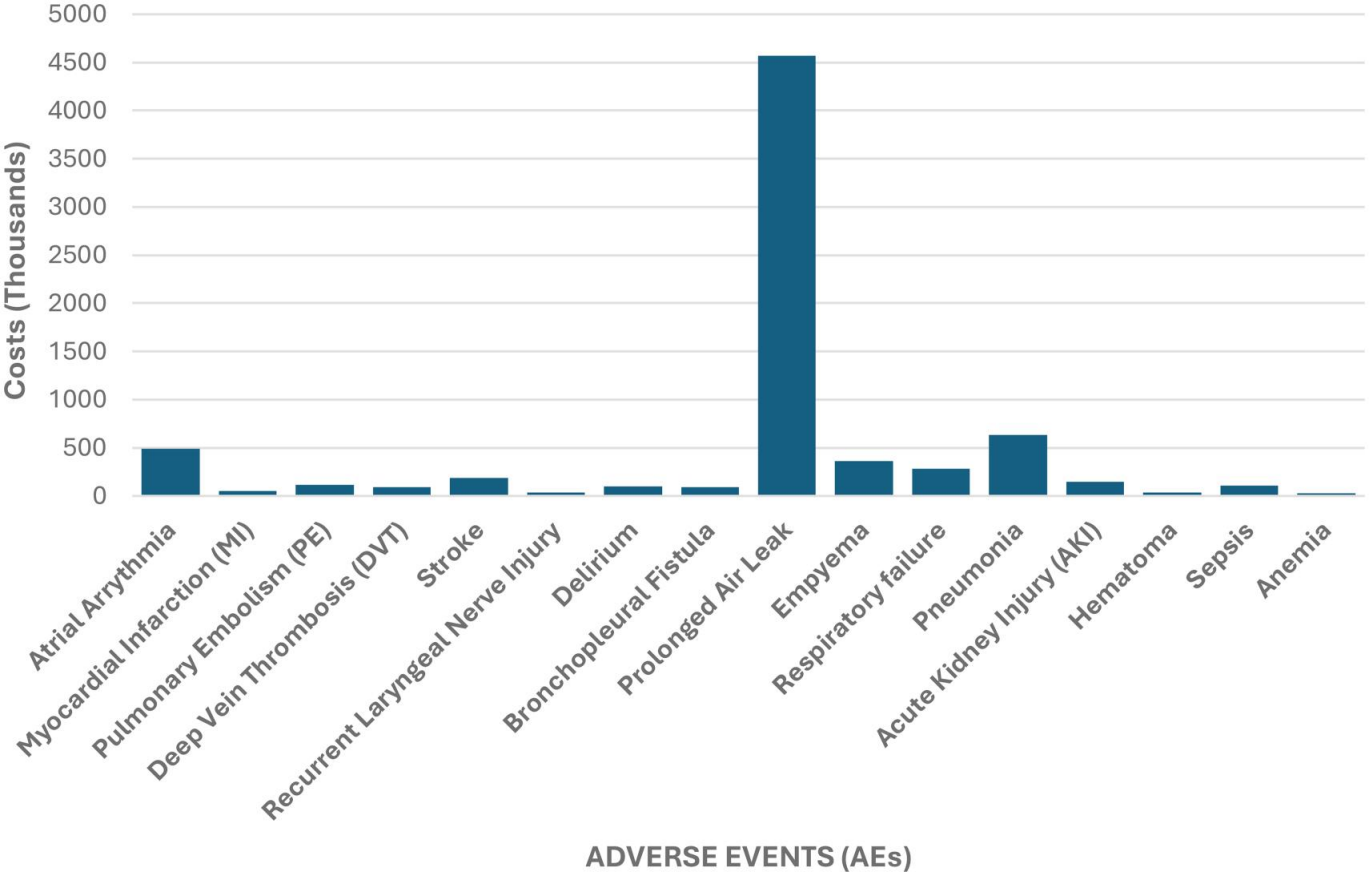
Graphical Representation of All Adverse Events (AEs) Across 10 Canadian High-Volume Thoracic Surgery Centres and Estimated Additional In-hospital Costs.

Data from 10 Canadian Lung Cancer centres (2017-2021) were included in our analysis. The mean annual hospital lobectomy volume was 1150 (SD = 165), and 44% of patients were male with a mean age of 67 (SD = 0.49). The median LOS was 4 days (IQR 2-6). Demographic information, along with the national mean incidence of AEs, characterized by system, is detailed in **Table 1**. **Table 2** depicts the incidence, SD, and CoV of all AEs across the 10 participating centres. **Table 3** depicts all AEs across the 10 participating centres, annual incidence, estimated cost per occurrence, and the estimated additional in-hospital costs. This amounted to cost estimates of over $7.31 million CDN annually to the Canadian healthcare system. **Figure 1** demonstrates the graphical representation of all AEs across the 10 participating centres. All AEs, including those with an incidence <2%, are reported to provide a comprehensive overview of complication-related cost patterns and to support benchmarking efforts across institutions.

**Table 1.** Volume and Patient Demographics of 10 High-Volume Canadian Thoracic Surgery Centres (Mean Across Study Years)

Volume and demographic information	Mean (u)	Standard deviation (SD)	Coefficient of variation (CoV, %)^a^
Annual lobectomy volume (N)	1150	165	14
Percentage male (%)	44	2.4	5
Age (years)	67	0.5	0.7
Length of stay (days)	4	[2-6]^b^	**—**
Minimally invasive surgery (MIS, %)	86	—	—

a CoV = coefficient of variation = SD ÷ Mean × 100.

b Interquartile range [IQR].

**Table 2.** Adverse Events (AEs) Following Lobectomy Using Thoracic Morbidity and Mortality (TM&M) System (Annual Means of 10 High-Volume Thoracic Centres)

Clavien-Dindo thoracic morbidity and mortality (TM&M) system	Adverse events (AEs)	Mean yearly incidence (%)	SD (%)	CoV (%)^a^
Cardiac	Atrial arrhythmia	5	11.7	21
	Myocardial infarction (MI)	2.1	1.7	69.3
	Pulmonary embolism (PE)	1	2.6	41.8
	Deep-vein thrombosis (DVT)	0.2	2.1	78.1
Neurologic	Stroke	1	1.3	26.5
	Recurrent laryngeal nerve (RLN) injury	0.3	2.6	72.4
	Delirium	1.5	4.8	33.5
Pleural	Prolonged air leak (PAL)	17	2.5	71.9
	Empyema	1	40.3	20.4
	Bronchopleural fistula (BPF)	0.3	3.6	36.4
Pulmonary	Pneumonia	3	5.5	46.2
	Respiratory failure	1	10.3	30.5
Renal	Acute kidney injury (AKI)	0.6	5.3	71.3
Wound	Haematoma	0.3	0.5	18.2
Other	Sepsis	0.2	1	40
	Anaemia	0.1	0.6	34.2

a CoV = coefficient of variation = SD ÷ Mean × 100.

**Table 3.** Adverse Events (AEs) Across 10 Canadian High-Volume Thoracic Surgery Centres and Estimated Additional In-hospital Costs

Clavien-Dindo thoracic morbidity and mortality (TM&M) system^a^	Adverse events (AEs)	Mean yearly incidence (%)	Estimated annual cost (CDN 2025)
Cardiac	Atrial arrhythmia	5	$490 000
	Pulmonary embolism (PE)	1	$117 000
	Myocardial infarction (MI)	0.2	$54 904
	Deep-vein thrombosis (DVT)	0.2	$89 039
Neurologic	Stroke	1	$185 400
	Recurrent laryngeal nerve (RLN) injury	0.31	$32 836
	Delirium	1.5	$102 300
Pleural	Prolonged air leak (PAL)	17	$4 600 000
	Empyema	1	$360 000
	Bronchopleural fistula (BPF)	0.3	$88 270
Pulmonary	Pneumonia	3	$630 708
	Respiratory failure	1	$283 601
Renal	Acute kidney injury (AKI)	0.64	$146 000
Wound	Haematoma	0.25	$34 694
Other	Sepsis	0.2	$108 130
	Anaemia	0.14	$25 162
	Estimated total cost: **$7.31 million CDN**		

a Seely AJ et al. Systematic classification of morbidity and mortality after thoracic surgery. Ann Thorac Surg. 2010 Sep; 90(3):936-42.

**Table 4** displays the 9 most common AEs, characterized by system, with accompanying AEs incident rates and estimated additional in-hospital costs. This includes cardiac events (atrial arrhythmia, 5% incidence rate, $489 613 CDN; pulmonary embolism [PE], 1%, $117 118 CND), neurological events (stroke, 1%, $185 373 CDN; delirium, 1.5%, $102 312 CND), pleural events (PAL, 17%, $4 566 651 CDN; empyema, 1%, $359 199 CDN), pulmonary events (respiratory failure, 1%, $283 601 CDN; pneumonia, 3%, $630 708), and renal events (acute kidney injury [AKI], 1%, $145 299 CDN). In total, these 9 AEs contribute approximately $6.9 million annually to the Canadian healthcare system. Furthermore, PAL, as a sole complication, drives 66% of these additional costs.

**Table 4.** 9 Most Common Adverse Events (AEs) Across 10 Canadian High-Volume Thoracic Surgery Centres and Estimated Additional In-hospital Costs

Clavien-Dindo thoracic morbidity and mortality (TM&M) system	Adverse events (AEs)	Mean yearly incidence (%)	Cost per occurrence (CDN 2025)	Estimated annual cost (CDN 2025)
Cardiac	Atrial arrhythmia	5	$8806	$489 614
	Pulmonary embolism (PE)	0.5	$18 890	$117 118
Neurologic	Stroke	1	$39 026	$185 374
	Delirium	1.5	$7105	$102 312
Pleural	Prolonged air leak (PAL)	17.2	$23 134	$4 566 652
	Empyema	1	$36 653	$359 199
Pulmonary	Pneumonia	3	$18 660	$630 708
	Respiratory failure	1	$24 034	$283 601
Renal	Acute kidney injury (AKI)	0.6	$19 635	$145 299

**Figure 2.**
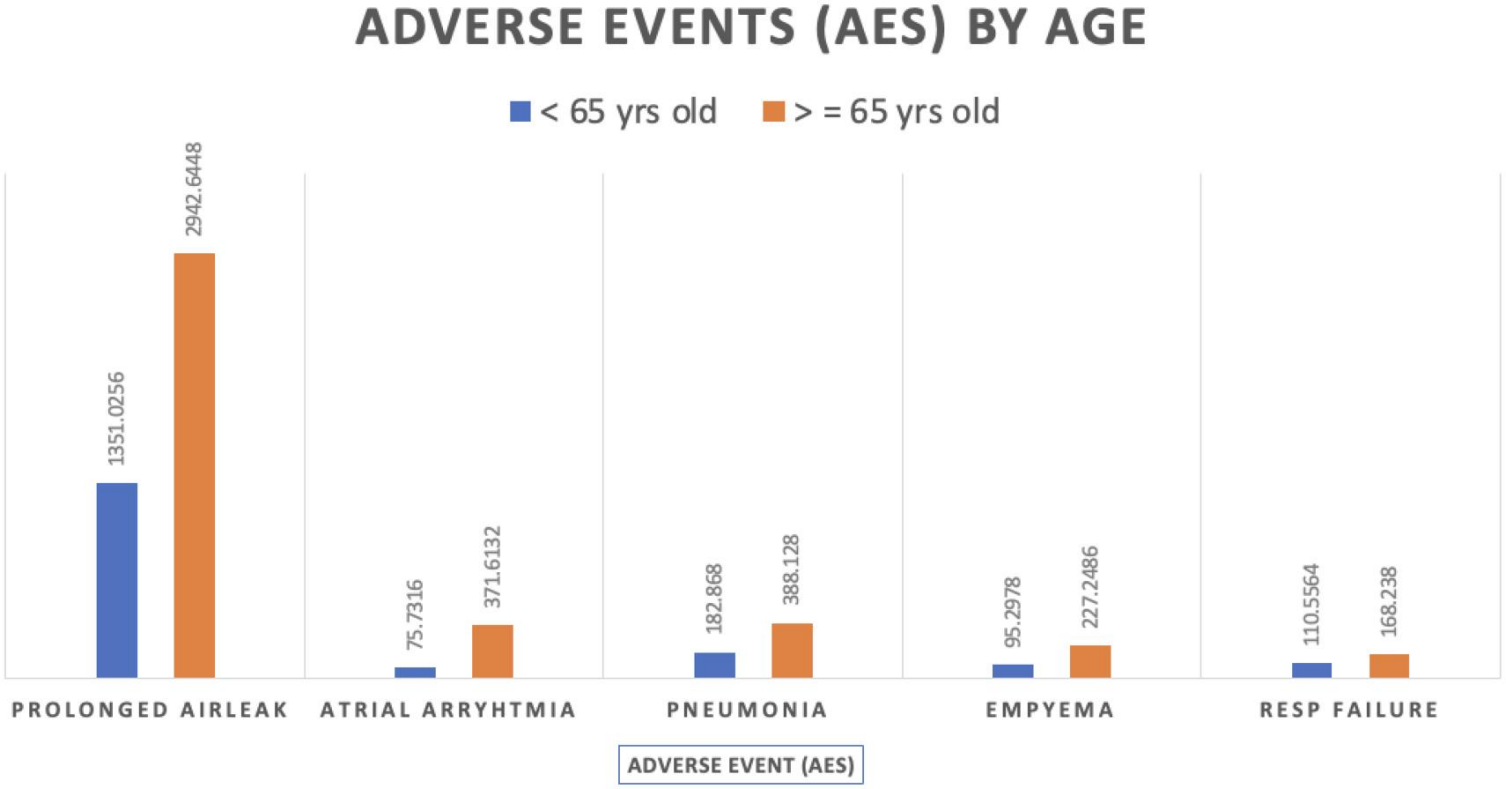
Cost Estimates of the 5 Most Expensive Adverse Events (AEs) by Age (65).

**Table 5** demonstrates the impact of age on cost of in-hospital complications. Patients under the age of 65 (< 65 years old) who experienced a PAL added an additional $1 351 025 CDN/year in-hospital costs, while patients aged ≥ 65 added $2 942 644 CDN/year to hospital costs. This increased cost ratio was revealed for atrial arrhythmia (<65: $75 732 CDN; ≥65 $371 613), pneumonia (<65: $182 868 CDN; ≥65: $388 128), empyema (<65: $95 298 CDN; ≥65: $227 249 CDN), and respiratory failure (<65: $110 556 CDN; ≥65: $168 238 CDN). **Figure 2** depicts the incidence and cost estimates of the 5 most expensive AEs by age (<65 vs ≥65).

**Table 5.** Adverse Events (AEs) Across 10 Canadian High-Volume Thoracic Surgery Centres and Estimated Additional In-hospital Costs Based on Age

Clavien-Dindo thoracic morbidity and mortality (TM&M) system	Adverse events (AEs)	<65 (*n* = 1768)		≥65 (*n* = 3321)	
		Annual incident rate (%)	Estimated annual cost	Annual incident rate (%)	Estimated annual cost
Cardiac	Atrial arrhythmia	2.4	$75 732	6.4	$371 613
	Myocardial infarction (MI)	0.3	$21 962	0.3	$49 415
	Pulmonary embolism (PE)	0.5	$37 780	0.7	$86 894
	Deep-vein thrombosis (DVT)	0.3	$33 348	0.2	$55 691
Neurologic	Stroke	0.6	$78 052	0.5	$126 835
	Recurrent laryngeal nerve (RLN) injury	0.5	$15 232	0.4	$23 715
	Delirium	0.9	$23 091	1.8	$82 418
Pleural	Prolonged air leak (PAL)	16.6	$1 351 026	19.2	$2 942 645
	Empyema	0.7	$95 298	0.9	$227 249
	Bronchopleural fistula (BPF)	0.4	$31 525	0.4	$75 660
Pulmonary	Pneumonia	1.3	$110 556	1.1	$168 238
	Respiratory failure	2.8	$182 868	3.1	$388 128
Renal	Acute kidney injury (AKI)	1.0	$72 060	0.8	$102 102
Wound	Haematoma	0.4	$15 770	0.3	$18 924
Other	Sepsis	0.4	$64 878	0.3	$75 691
	Anaemia	0.4	$15 726	0.2	$22 016

### Impact across Canada

Between 2017 and 2022, on mean 7522 lobectomies were performed annually in Canada.^10^ Our historical dataset with prospective AE collection contained data on 1150 lobectomies for cancer, with an estimated additional in-hospital costs of $7.31 million, amounted to approximately $48 million across Canada yearly. If hospitals can produce an absolute reduction (AR) in the 4 most common AEs (ie, delirium, pneumonia, PAL, and atrial fibrillation) by 1%, this would result in hospital level cost savings of over $4.3 million CDN/year. Similarly, an AR of 3% of these same AEs would result in cost savings of more than $15 million CDN/year.

## Discussion

The annual in-hospital cost of AEs following lobectomy across 10 Canadian centres is estimated at $7.31 million CAD. When extrapolated nationally, this figure exceeds $48 million CDN per year, representing a significant burden to hospitals, the healthcare system, and society. To our knowledge, this is the first study to quantify the increased Canadian hospitalization costs attributable to lobectomy-related AEs. To contextualize these projections, annual Canadian health spending is approximately $344 billion CAD (2023), meaning that a reduction of up to $15 million CAD per year in lobectomy-related AE costs represents a small but meaningful efficiency gain within thoracic surgery, particularly given that these reductions stem from preventable and targeted perioperative improvements. These findings likely underestimate the true financial and human impact, as indirect costs—such as lost productivity due to morbidity or premature mortality—are not included. As lung cancer remains the leading cause of cancer-related death in both men and women, addressing the hospital-level costs of AEs is a critical target for future QI initiatives. From a health policy perspective, the potential cost savings associated with even small reductions in AE rates are substantial. In our cohort, a 1% absolute reduction in the 4 most common complications—delirium, pneumonia, PAL, and atrial fibrillation—would result in estimated savings of over $4.3 million CDN annually at the hospital level. A 3% reduction would yield more than $15 million CDN in national savings each year. These figures illustrate the significant financial leverage of QI initiatives in thoracic surgery, where relatively modest improvements in complication rates can yield disproportionately large cost reductions. Such projections reinforce the importance of investing in perioperative optimization, standardized care pathways, and data-driven interventions as part of a national value-based healthcare (VBHC) strategy.

A multipronged strategy is essential to understand and address complications following lobectomy and to translate insights from the costs of AEs into improved patient outcomes. Data-driven approaches that systematically analyse high-cost, high-impact AEs at the institutional level can reveal actionable patterns, patient-specific risk factors, and preventive strategies aimed at reducing both clinical and economic consequences.^11^ Comprehensive preoperative risk assessments are critical for identifying patients at elevated risk, enabling tailored perioperative care—an approach supported by evidence from large database studies.^12^ Enhanced preoperative planning, particularly in frail populations, plays a pivotal role in mitigating postoperative risks and reducing AE incidence.^13^ Standardizing perioperative protocols further reduces practice variability, a known contributor to higher complication rates.^14^ In parallel, integrating real-time monitoring and early intervention strategies—potentially powered by artificial intelligence—may facilitate the early detection of clinical deterioration, allowing for timely interventions that prevent escalation of complications. However, while such broad system-level initiatives can improve overall perioperative outcomes, the reduction of PAL—the most frequent and costly AE—requires focused, evidence-based strategies. Technical measures play a pivotal role, including careful fissure dissection or fissureless lobectomy techniques,^15^ and selective use of surgical sealants or buttressing for patients with incomplete fissures or emphysematous lungs.^16^ Perioperative factors, such as lung-protective ventilation strategies and judicious chest tube management, also influence air-leak duration. Patient-related risk stratification remains essential, as reduced pulmonary reserve, low BMI, and steroid use are established predictors of PAL.^17^^,^^18^ Integrating these strategies into existing perioperative pathways may yield the most meaningful reduction in PAL incidence and associated costs. Future QI efforts should therefore focus on both surgical technique optimization and standardized postoperative management.

Comparable cost analyses from the United States and Europe confirm a disproportionate financial burden of postoperative AEs following lobectomy. For instance, a US-based study demonstrated the mean cost increment of lobectomy associated AEs was ∼22% for PAL and ∼96% for pneumonia.^19^ In Europe, a UK-based study found that lobectomy patients with PAL incurred mean postoperative costs of ∼US$5939 compared with ∼US$4382 for those without PAL.^20^ Another European centre estimated additional treatment costs for PAL of ∼€2888 to €12 343.^21^ Although differences in health‐care system structure and cost accounting limit direct dollar-to-dollar comparisons, the relative magnitude and key cost drivers (particularly PAL, pneumonia, and arrhythmia) show strong alignment across geographies. These parallel studies support the generalizability of our Canadian-focused findings and underscores the global priority of targeting high‐cost thoracic-surgery AEs.

Understanding the cost of postoperative AEs is a critical factor in the shift towards VBHC.^22^ AEs not only drive direct costs, and likely substantially increase indirect costs, they also negatively impact patient outcomes and satisfaction.^23^ The high inter-institutional variability observed in our present study (reflected by large CoV values) suggests that AE rates and associated costs are not uniformly distributed across centres. From a VBHC perspective, this heterogeneity implies that a uniform allocation of resources may not be optimal. Instead, targeted investment in centres with higher AE rates—through enhanced recovery programmes, staff training, or perioperative monitoring—may yield greater system-wide value. Furthermore, by identifying high-performing centres (through positive deviance [PD] seminars) and disseminating their effective practices, this could further reduce variation and promote cost-efficient, high-quality care delivery. In thoracic surgery, where institutional and surgeon-level variation in perioperative practices remains, PD seminars can uncover nuanced, effective techniques that are already yielding better results in peer institutions.^24–26^ Ultimately, such an approach aligns with the principles of VBHC, emphasizing outcome improvement per dollar spent while recognizing that the marginal benefit of resource deployment may be highest in institutions with the greatest potential for improvement.

The observed increase in AE-related costs among patients aged ≥65 years reflects a combination of clinical and systemic factors. Older adults often present with frailty, reduced physiological reserve, and multiple comorbidities, predisposing to severe or concurrent postoperative complications. These patients also tend to require intensive monitoring, additional interventions, and more discharge coordination, all of which amplify hospital resource use. Furthermore, delayed recovery and discharge planning—often related to functional decline or the need for post-hospitalized care—can extend LOS and compound total costs. Understanding these multifactorial contributors underscores the importance of preoperative geriatric assessment and tailored perioperative management for older patients undergoing lobectomy.

Our study has several limitations. First, our cost estimation method has structural limitations. We relied on literature-derived estimates of index hospitalization costs to quantify the financial burden of AEs following lobectomy. As cost estimates were derived using an event-based approach, patients experiencing multiple complications may have contributed to more than one AE category, leading to potential overestimation of cumulative costs. However, our intent was to describe the relative financial burden of individual complications rather than the exact per-patient cumulative cost. AE-related costs are multifaceted—encompassing direct medical expenses, reoperations, medication use, and intensive care utilization—making it likely that our findings underestimate the true economic impact. As such, the results should be interpreted as AE-specific cost burden estimates intended to inform prioritization rather than as precise system-wide expenditure totals. A more accurate assessment requires institution-specific, data-driven cost analyses, which remain the focus of our ongoing research. Notably, over half of the cost studies included in our analysis (11 of 20) originated from the United States, with none based in Canada, thereby limiting the generalizability of our findings to the Canadian healthcare context and potentially introducing bias in international comparisons. Moreover, our analysis was confined to in-hospital costs during the index admission and did not capture post-discharge or indirect societal costs—such as prolonged recovery, productivity loss, and long-term rehabilitation—which are likely substantial. Accurately estimating potential cost savings and informing strategies to improve clinical outcomes requires detailed institutional data, supported by standardized reporting of AE incidence and severity. Although the CATS database applies a standardized definition of PAL (>5 postoperative days), differences in postoperative management—such as the use of digital drainage systems, criteria for chest tube removal, and indications for pleurodesis—may influence its reported incidence. Similarly, the absence of uniformly collected comorbidity and procedural detail data—such as COPD staging, smoking status, and resection extent—within the current CATS dataset may partly explain inter-institutional variation in complication rates and costs. Future research must prioritize multicentre cost analyses that incorporate comorbidity burden and procedural complexity, as well as patient-reported outcome measures, long-term follow-up, and national policy perspectives. Such standardization would help fully capture the downstream impact of lobectomy-related complications, enabling benchmarking across institutions and supporting more nuanced cost evaluations. Finally, our cost model assumes independence between complications, meaning that patients experiencing multiple AEs contribute to several AE-specific cost categories. This event-based approach may modestly inflate cumulative cost estimates. In addition, our study is a descriptive economic evaluation based on an event-level cost model, rather than a patient-level cost analysis. Because literature-derived AE cost estimates are applied uniformly to aggregated AE counts rather than to individual patient cost data, inferential statistical testing—including confidence intervals around cost estimates or formal hypothesis tests comparing subgroups—is not statistically valid in this context. Similarly, incidence differences across institutions or age strata are descriptive and not adjusted for patient-level covariates. Our findings therefore should be interpreted as population-level cost estimates intended to identify high-burden AEs and guide quality-improvement prioritization.

## Conclusions

In summary, the estimated additional in-hospital costs associated with lobectomy-related AEs from 10 high-volume Canadian centres exceed $7.31 million CAD annually. When extrapolated to a national scale, these complications impose a financial burden of over $48 million CAD per year on the Canadian healthcare system and taxpayers. Addressing the cost of complications is essential for delivering high-value care, improving clinical outcomes, and ensuring the long-term sustainability of the healthcare system. Future research should prioritize the evaluation of long-term and indirect societal costs—such as productivity loss and prolonged recovery—which are likely substantial. These data will also enhance the informed consent process by allowing patients with lung cancer to better understand the potential risks and downstream consequences of surgery.

## Data Availability

The data underlying this article are available within the article and upon request.
